# More than just a side effect: Dynamic knee valgus and deadbug bridging performance in youth soccer players and alpine skiers have similar absolute values and asymmetry magnitudes but differ in terms of the direction of laterality

**DOI:** 10.3389/fphys.2023.1129351

**Published:** 2023-03-08

**Authors:** Jonas Hanimann, Lynn Ellenberger, Thomas Bernhard, Martino V. Franchi, Ralf Roth, Oliver Faude, Jörg Spörri

**Affiliations:** ^1^ Sports Medical Research Group, Department of Orthopaedics, Balgrist University Hospital, University of Zurich, Zurich, Switzerland; ^2^ University Centre for Prevention and Sports Medicine, Department of Orthopaedics, Balgrist University Hospital, University of Zurich, Zurich, Switzerland; ^3^ Swiss Council for Accident Prevention BFU, Bern, Switzerland; ^4^ Department of Sport, Exercise and Health, University of Basel, Basel, Switzerland; ^5^ FC Basel 1893, Basel, Switzerland; ^6^ Institute of Physiology, Department of Biomedical Sciences, University of Padova, Padova, Italy

**Keywords:** athletes, alpine skiing, soccer, performance, injury prevention, exercise test

## Abstract

From a preventative perspective, leg axis and core stabilization capacities are important for soccer players and alpine skiers; however, due to different sport-specific demands, the role of laterality clearly differs and may result in functional long-term adaptations. The aims of this study are 1) to determine whether there are differences in leg axis and core stability between youth soccer players and alpine skiers and 2) between dominant and non-dominant sides, and 3) to explore the outcomes of applying common sport-specific asymmetry thresholds to these two distinct cohorts. Twenty-one highly trained/national-level soccer players (16.1 years, 95% CI: 15.6, 16.5) and 61 alpine skiers (15.7 years, 95% CI: 15.6, 15.8) participated in this study. Using a marker-based 3D motion capture system, dynamic knee valgus was quantified as the medial knee displacement (*MKD*) during drop jump landings, and core stability was quantified as the vertical displacement during deadbug bridging exercise (*DBB*_*displacement*_). For the analysis of sports and side differences, a repeated-measures multivariate analysis of variance was used. For the interpretation of laterality, coefficients of variation (CV) and common asymmetry thresholds were applied. There were no differences in *MKD* or *DBB*_*displacement*_ between soccer players and skiers or between the dominant and non-dominant sides, but there was an interaction effect side*sports for both variables (*MKD*: *p* = 0.040, η^2^ *p* = 0.052; *DBB*_*displacement*_: *p* = 0.025, η^2^ *p* = 0.061). On average, *MKD* was larger on the non-dominant side and *DBB*_*displacement*_ laterality on the dominant side in soccer players, whereas this pattern was reversed in alpine skiers. Despite similar absolute values and asymmetry magnitudes of dynamic knee valgus and deadbug bridging performance in youth soccer players and alpine skiers, the effect on the direction of laterality was opposite even though much less pronounced. This may imply that sport-specific demands and potential laterality advantages should be considered when dealing with asymmetries in athletes.

## 1 Introduction

Humans typically prefer one side for the execution of motor tasks, hence resulting in a more skilled side, often called laterality or side dominance (Carpes et al., 2010; Maloney, 2019; Hart et al., 2020; Westin et al., 2022b). In this context, laterality describes a difference in body morphology or function (Hart et al., 2020). Several sports-related motor tasks, such as throwing or kicking, are strongly related to unilateral execution, thus manifesting in a high degree of laterality as a consequence of functional long-term adaptations (Newton et al., 2006; Parrington and Ball, 2016; Bishop et al., 2018b; Lijewski et al., 2021), while others are more symmetrical with less pronounced, but still present, laterality (e.g., running, cycling, or swimming) (Parrington and Ball, 2016; Maloney, 2019). One sport with high laterality is soccer, a globally popular team sport where physical, tactical, and technical components are crucial for team success (Stolen et al., 2005). In terms of physical demands, well-performed changes of direction are the key and have been described as the best discriminant variable among young players regarding selection into superior teams (Gil et al., 2007a; Gil M. et al., 2007b). Although fast changes of direction in soccer are made as a reaction to game situations and, therefore, are multidirectional, the dominant leg can usually perform faster change of direction maneuvers than the non-dominant leg (Rouissi et al., 2016). Another central aspect in soccer is kicking, with players favoring one limb, which is referred to as the dominant limb (DeLang et al., 2019). In total, more than 80% of the ball contacts are performed with the dominant leg (Carey et al., 2001). Hence, soccer activity is strongly characterized by the asymmetrical nature of the sport. By contrast, for athletes from alpine skiing, i.e., a more symmetric sport, less laterality can be expected. Alpine skiing places high loads on the athletes’ bodies, forces up to 1.75 times their body weight on each leg (Kröll et al., 2016), combined with high knee flexion angles and valgus (Zorko et al., 2015), and enormous force and stabilization capacity are required. The interaction with snow causes vibration loads and impact-like shocks that are largely absorbed by the knee and its stabilizing muscles (Spörri et al., 2017; Supej et al., 2018). When compared to soccer, however, the physical demands are more symmetric, and thus, side dominance may play a subordinate role. Such different demands are likely to result in functional long-term adaptations that have to be considered when dealing with asymmetries in athletes.

Injuries are common in soccer players (Eirale et al., 2013; Hagglund et al., 2013). Anterior cruciate ligament (ACL) injury incidences are high (0.06–3.7 per 1,000 h of training and competition) (Bjordal et al., 1997; Fauno and Wulff Jakobsen, 2006) and predominantly occur without player-to-player contact; hence, ACL injuries are referred to as non-contact injuries (Fauno and Wulff Jakobsen, 2006; Alentorn-Geli et al., 2009). Interestingly, the dominant leg, when compared to the non-dominant leg, is commonly associated with a decreased knee flexor (i.e., hamstrings) to knee extensor (i.e., quadriceps) strength ratio (Rahnama et al., 2005) and is more frequently subjected to injuries (DeLang et al., 2021). Typical situational patterns leading to ACL injuries are indirect or non-contact situations, such as pressing/tackling, regaining balance after kicking, landing from a jump, and being approached (Della Villa et al., 2020). In this regard, the following movements are at high risk: cutting maneuvers combined with deceleration, near- or fully extended jump landings, and pivoting with an extended knee and a planted foot (Boden et al., 2000; Fauno and Wulff Jakobsen, 2006). Similarly, alpine skiers are also at high risk of sustaining knee injuries (Florenes et al., 2009; Florenes et al., 2012), whereas ACL rupture represents the most frequent diagnosis (Florenes et al., 2009). ACL injuries almost exclusively occur while skiing; they are only rarely caused by a crash (Bere et al., 2011). In this regard, the following three main injury mechanisms are defined and described in detail elsewhere: slip-catch, landing back-weighted, and dynamic snowplow (Bere et al., 2011). In brief, when turning, the skier gets out of balance before the sudden edge catch of the outside ski forcing the knee into valgus and internal rotation (slip–catch mechanism) (Bere et al., 2011). Back-weighted jump landing results from losing balance during flight and then trying to recover, when a combination of tibiofemoral compression and anterior drawer of the tibia in relation to the femur acts on the athlete’s knee (Meyer and Haut, 2008; Bere et al., 2011). Dynamic snowplowing starts in a back-weighted out-of-balance situation, leading to a split position and unloaded outer ski, and the loaded ski grips on the inner edge, subsequently leading to internal rotation and valgus (Bere et al., 2011).

Considering the aforementioned sport-specific mechanisms of ACL injuries and concomitant lesions, it is reasonable to consider poor leg axis stability (i.e., extensive dynamic knee valgus) a modifiable risk factor (Hewett et al., 2006) that plays a key role in injury mechanisms (Bere et al., 2011). Medial knee displacement (*MKD*) can be reliably quantified during drop jump (DJ) landing (Paterno et al., 2010; Krosshaug et al., 2016; Leppanen et al., 2016; Ellenberger et al., 2020b). Furthermore, core stability may be preventative in the context of out-of-balance mechanisms in ACL injuries (Hewett et al., 2005; Zazulak et al., 2007). However, an objective, reliable, and valid quantification of core stability is challenging (Hibbs et al., 2008). A more holistic approach that has been proven to be suitable in the context of injury prevention is the quantification of the rear chain stabilization capacity (Ellenberger et al., 2020a). In this regard, the anti-torsional stabilization capacity is assessed through the hip axis tilt in the frontal plane during deadbug bridging (*DBB*_*displacement*_) (Ellenberger et al., 2020a). Both the *MKD* during DJ landings and *DBB*_*displacement*_ are preferably quantified with a motion capture system, such as the Vicon Nexus.

To what extent does high degrees of laterality influence athletic performance and the risk of injury in different sports has not yet been conclusively clarified (Knapik et al., 1991; Bourne et al., 2015; Bishop et al., 2018b; Dos'Santos et al., 2019; Maloney, 2019; Bishop et al., 2022; Westin et al., 2022a). Traditionally, subjects with interlimb asymmetries of >10%–15% have been associated with higher injury incidences than those with such asymmetries below this threshold (Barber et al., 1990; Impellizzeri et al., 2007; Grindem et al., 2011). However, it is not *a priori* clear whether, especially in highly asymmetrical sports, a high degree of laterality is adverse and must be prevented or whether, on the contrary, it is actually the key in enhancing performance or protecting athletes. Accordingly, recent research have proposed a more sophisticated approach for laterality analysis, which include cohort- and task-specific thresholds to account for the task- and metric-dependent nature of asymmetries (Bishop et al., 2018a; Bishop et al., 2021; Dos’Santos et al., 2021). Moreover, Exell et al. (2012) have stated that the asymmetry percentage must be larger than the coefficient of variation and suggested the application of an individual approach in the context of interlimb asymmetry considerations.

Accordingly, the aims of this study were threefold: 1) to determine whether there are distinct differences in leg axis and core stability between soccer players and skiers, 2) to investigate whether youth soccer players and alpine skiers exhibit leg axis and core stability differences between the dominant and non-dominant sides, and 3) to explore the outcomes of applying common sport-specific asymmetry thresholds to these two distinct cohorts. Considering the literature and the great importance of core and leg axis stability in both alpine skiing and soccer, similar absolute values have been hypothesized for both cohorts in their respective exercise tests. However, due to the asymmetric nature of the demands in soccer, which are in contrast to alpine skiing, it was been hypothesized that there would be greater differences between the dominant and non-dominant sides than it is for alpine skiers. Accordingly, a higher percentage of individuals in the soccer cohort were expected to be classified as asymmetric.

## 2 Materials and methods

### 2.1 Participants and study design

Twenty-one male youth soccer players and 61 youth alpine skiers participated in a cross-sectional study and were assessed with respect to leg axis and core stability as further defined below. All data were collected at a single point in time [i.e., before the competitive season in October (alpine skiers) and in January (soccer players)] and were analyzed without any interventional influence. Data collection took place on dedicated test days directly at the athletes’ training facilities using a standardized mobile measurement setup and operated by the same experienced team of evaluators. Standard pretest preparation advice included no intense training or competition 24 h prior to testing and only healthy athletes participated.

Alpine skiers were recruited through their membership in a youth development structure of a national skiing association. The recruitment of the soccer players was based on their membership in a professional youth soccer academy and playing in the corresponding U16–U18 teams. Regarding training and performance classification, both cohorts met the criteria for *Tier 3*, which is defined as highly trained and competing at the national level (McKay et al., 2022). Further eligibility criteria were not being in a back-to-sports program after injury and not having systematic pathologies, diabetes mellitus, or inflammatory arthritis. The resulting sample size represents the full availability of healthy athletes within the cooperating associations at the time of assessment.

The selection of the two sports investigated was based, as outlined in the introduction, on the idea of comparing a group with highly symmetrical sport-specific requirements with a group that particularly has asymmetrical requirements. Alpine skiing and soccer are the two sports that fulfil this criterion and are also of high interest for injury prevention research due to their high risks.

All participants/participants’ legal guardian/next of kin were informed about the study and provided written informed consent. The corresponding study protocols were approved by the local ethics committees (KEK-ZH-NR: 2017-01395 and EKNZ 2017-02148), and the procedures were in full accordance with the Declaration of Helsinki and national laws.

### 2.2 Data collection

Leg axis stability was quantified as medial knee displacement (*MKD*; in mm) during drop jump (DJ) landings (Ellenberger et al., 2020b). The *MKD* was specified as the maximal distance between the knee joint center during the ground contact phase and the predefined reference plane. The reference plane consisted of the hip, knee joint center, and ankle joint center and was set to one frame before ground contact. A threshold of 25 N was used to determine ground contact for both legs independently. The subjects were instructed to drop off from a 32-cm-high box in upright posture and subsequently perform a maximum height vertical jump with minimal ground contact time. Throughout the trial, both hands had to remain on the pelvis, and the subjects had to land with their feet on two adjacent force plates. A trial was considered invalid if the participants i) actively jumped off the box, ii) lost hand contact with the pelvis, iii) did not correctly hit the force plates, or iv) had hesitation in jumping off after landing. The subjects were asked to perform additional trials until two valid trials were recorded, with a minimum of 15 s of recovery time between the trials.

Core stability was quantified as the maximum amplitude of the vertical displacement (in mm) of the two pelvis markers during deadbug bridging exercise, with the marker of the stabilizing side representing the reference marker (*DBB*_*displacement*_), as suggested previously (Ellenberger et al., 2020a). Thus, *DBB*_*displacement*_ of the dominant side represents the displacement with the dominant side stabilizing while the non-dominant leg is lifted, and *vice versa*. In this regard, the subjects were asked to take a supine position on the floor with their arms abducted 90° from the body and their palms facing upward. Leg abduction was oriented such that the heels were in line with the elbows. To reach the starting position, the athletes were asked to lift their hip and keep ground contact only with their heels and shoulders. Subsequently, one heel had to be lifted to a position with knee and hip flexion angles of 90°. Holding this position for 3 s and returning to the starting position was one repetition. One trial consisted of three consecutive repetitions, without the hip touching the ground in between. The trial was repeated if i) the system could not detect the markers properly due to hip flexion, ii) the starting position was not taken properly, or iii) the hip touched the ground.

Biomechanical assessments were recorded with an optoelectronic 3D motion capture system with eight cameras (Vicon, Oxford Metrics) operating at 200 Hz. Additionally, two force plates were included in and synchronized with the measurement setup (SP Sportdiagnosegeräte GmbH) operating at 2,000 Hz. Participants were equipped with 31 reflective skin markers for DJ assessment, and placement was performed as defined by (Ellenberger et al., 2020b) in a slightly modified form of the plug-in-gait model (Vicon Nexus v2.6, Oxford Metrics). Prior to the dynamic assessment, four additional markers were placed on the medial femur epicondyles and the medial malleoli on both legs, and a static calibration was performed, allowing a more precise determination of knee and ankle joint centers. For the deadbug bridging performance assessments, four markers were bilaterally placed on the anterior superior iliac spine and the lateral malleoli. For both groups, the dominant leg was defined as the preferred leg to perform a soccer kick. All assessments were performed barefoot.

### 2.3 Data evaluation

Marker trajectories were identified using the Vicon Nexus software (Vicon Nexus v2.6, Oxford Metrics). Subsequently, the data were transferred to MATLAB (MATLAB R2016b, MathWorks, Inc.), and a customized MATLAB script was used for post-processing and parameter calculation. Interpolation of gaps in the marker trajectory was performed for a maximum of 10 frames (0.05 s). For DJ data processing, the reference plane was set one frame before ground contact for each leg separately and remained fixed at the hip joint center throughout the contact phase. The rectangular distance between the reference plane and the knee joint center throughout the trial was considered in *MKD* [mm]. Deadbug bridging trials were cut into three repetitions, identified through the minimal vertical height of the lateral malleolus marker of the lifted leg. For the three repetitions, the maximal amplitude in millimeters of the vertical displacement of the anterior superior iliac spine markers was averaged and then considered *DBB*_*displacement*_, with the height of the stabilizing side as the reference. Such protocols for assessing *MKD* and *DBB*_*displacement*_ have been shown to be reliable in previous studies (Ellenberger et al., 2020a; Ellenberger et al., 2020b). Individual laterality assessments were performed following the suggestions of Impellizzeri et al. (2007); asymmetry was thus calculated as the difference between the larger and smaller values divided by the larger value and represented in percent (Impellizzeri et al., 2007). To make visible which side had larger displacement, all asymmetry values where the non-dominant side represented the larger displacement value were multiplied by −1.

$$Asymmetry\left(\%\right)=\frac{larger\;displacement\;value-smaller\;displacement\;value}{larger\;displacement\;value}\times 100$$

### 2.4 Statistical analysis

The IBM SPSS statistics software version 28 was used for statistical analysis. The assumption of normality was checked for all metric data using the Shapiro‒Wilk test. All baseline characteristics are expressed as the group mean with 95% confidence interval in brackets. Repeated-measures multivariate analysis of variance (MANOVA) with Bonferroni correction for pairwise comparisons was used for the analysis of potential *MKD* and *DBB*_*displacement*_ differences. The within-subject factor was the side (dominant *vs*. non-dominant), and the between-subject factor was sports (soccer *vs*. alpine skiing). For the interpretation of laterality, coefficients of variation (CVs) and common asymmetry thresholds were used (Impellizzeri et al., 2007; Exell et al., 2012; Bishop et al., 2018a; Dos’Santos et al., 2021). In brief, the CV was calculated for each subject and exercised individually as a measure of reliability, and asymmetry thresholds were calculated for both cohorts and exercises as the classification criteria for the distinctiveness of laterality. Small to moderate asymmetry was assumed when athletes were above a threshold calculated as population mean + smallest worthwhile change (SWC; defined as 0.2 * SD between subjects) (Dos’Santos et al., 2021). The high asymmetry threshold was defined as laterality differences above the population mean + SD (Dos’Santos et al., 2021).

## 3 Results

### 3.1 Baseline characteristics

The baseline characteristics for all participating soccer players and alpine skiers, such as age, body height, and body weight, are presented in Table 1.

**TABLE 1:** Baseline characteristics.

	Soccer players (*n* = 21)	Alpine skiers (*n* = 61)
Age [years]	16.1 (15.6, 16.5)	15.7 (15.6, 15.8)
Body height [cm]	175.5 (173.2, 177.9)	172.4 (170.6, 174.3)
Body weight [kg]	66.0 (62.5, 69.4)	62.8 (60.3, 65.3)

Data are expressed as the group mean with 95% confidence intervals (CIs) in brackets.

### 3.2 Repeated-measures multivariate analysis of variance

**FIGURE 1:**
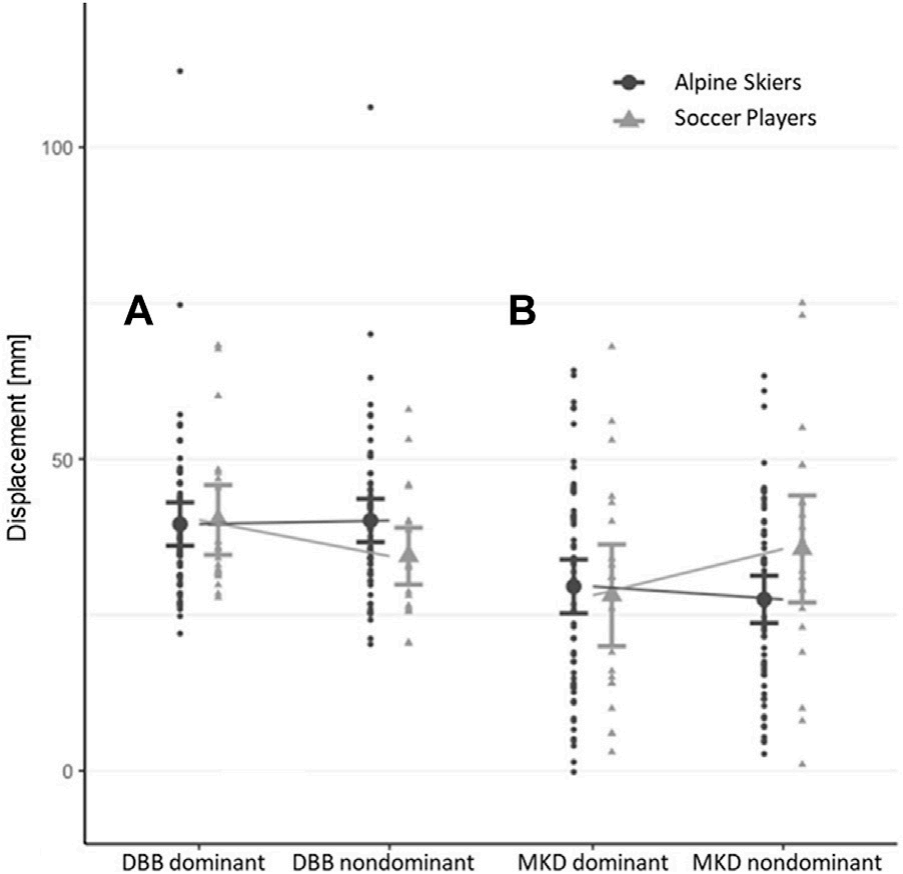
**(A,B)** Diagram with population mean, 95% CI, and individual values for deadbug bridging (DBB) and medial knee displacement (MKD). **(A)** Profile diagram for the interaction effect side*sports with respect to the maximum amplitude of the vertical displacement of the two pelvis markers during DBB exercise, with the marker of the stabilizing side representing the reference marker (DBB_displacement_). **(B)** Profile diagram for the interaction effect side*sports withrespect to MKD.

On a multivariate level, there were no significant differences between the sports (soccer player *vs*. skiers; *p* = 0.459, η^2^ *p* = 0.020) and the side (dominant *vs*. non-dominant; *p* = 0.107, η^2^ *p* = 0.055), but there was an interaction effect side*sports (*p* = 0.014, η^2^ *p* = 0.102). As presented in Figures 1A, B, univariate tests did not reveal any significant differences in *MKD* (*p* = 0.345, η^2^ *p* = 0.011) or *DBB*_*displacement*_ (*p* = 0.398, η^2^ *p* = 0.009) between the sports or between sides (*MKD*: *p* = 0.244, η^2^ *p* = 0.017; *DBB*_*displacement*_: *p* = 0.065, η^2^ *p* = 0.042), but an interaction effect side*sports was observed for both variables (*MKD*: *p* = 0.040, η^2^ *p* = 0.052; *DBB*_*displacement*_: *p* = 0.025, η^2^ *p* = 0.061) (Table 2).

**TABLE 2:** Medial knee displacement (MKD) and deadbug bridging displacement (DBB) for soccer players and alpine skiers.

	Soccer players (*n* = 21)	Alpine skiers (*n* = 61)
DBB dominant [mm]	40 (35, 46)	40 (36, 43)
DBB non-dominant [mm]	34 (30, 39)	40 (37, 44)
MKD dominant [mm]	28 (20, 36)	30 (25, 34)
MKD non-dominant [mm]	36 (27, 44)	28 (24, 31)

Data are expressed as the group mean with 95% confidence intervals (CIs) in brackets.

A detailed overview of the *MKD* and *DBB*_*displacement*_ values for each side and sport is given in Figure 1. On average, *MKD* laterality was directed to the non-dominant side and *DBB*_*displacement*_ laterality to the dominant side in soccer players, whereas this pattern was reversed in alpine skiers, even though it was much less pronounced. Thus, despite similar absolute values and asymmetry magnitudes of dynamic knee valgus and deadbug bridging performance in youth soccer players and alpine skiers, the effect on the direction of laterality was opposite.

### 3.3 CV values and derivation of sport-specific asymmetry thresholds

**FIGURE 2:**
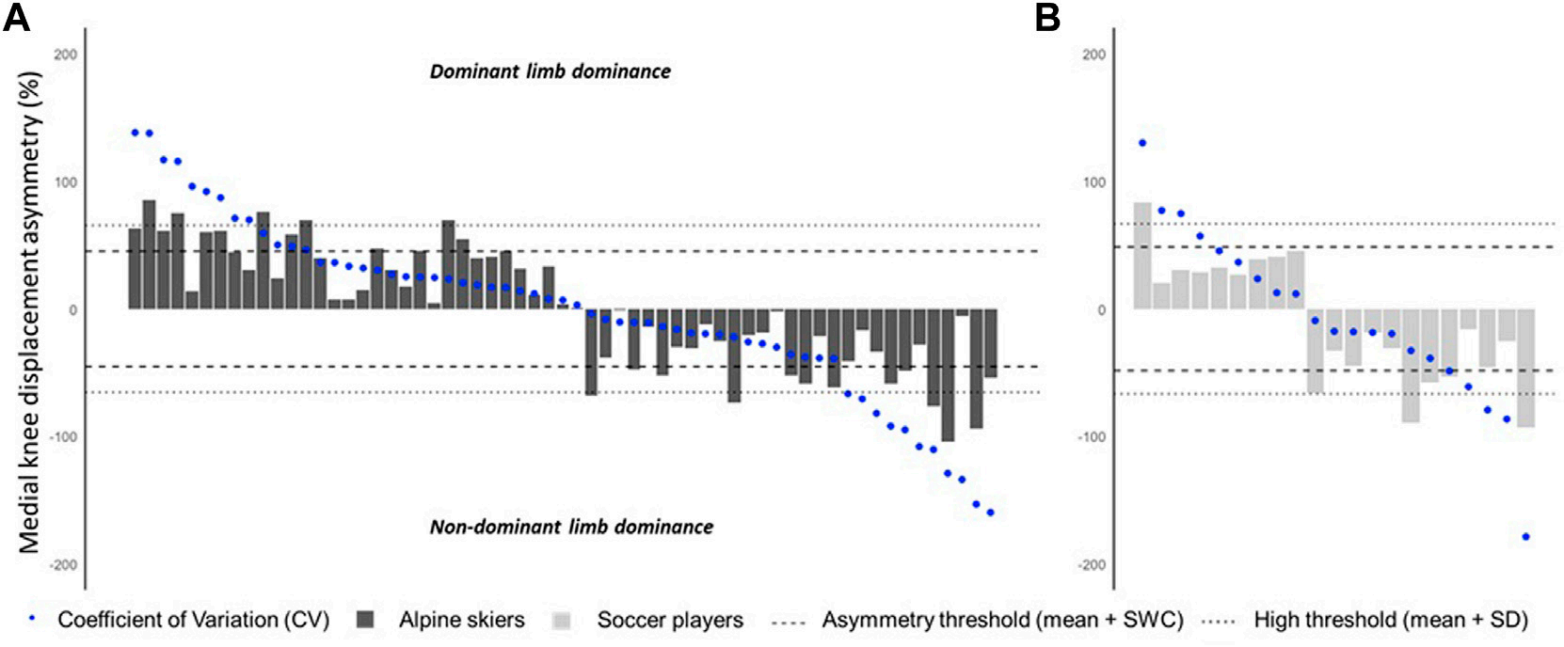
**(A,B)** Individual medial knee displacement (MKD) asymmetry data (bars) with the respective coefficient of variation (blue bullet points), threshold for small to moderate asymmetry [population mean + smallest worthwhile change (SWC), dashed lines], and threshold for high asymmetry (population mean + SD, dotted lines). **(A)** Alpine skiers; **(B)** soccer players.

**FIGURE 3:**
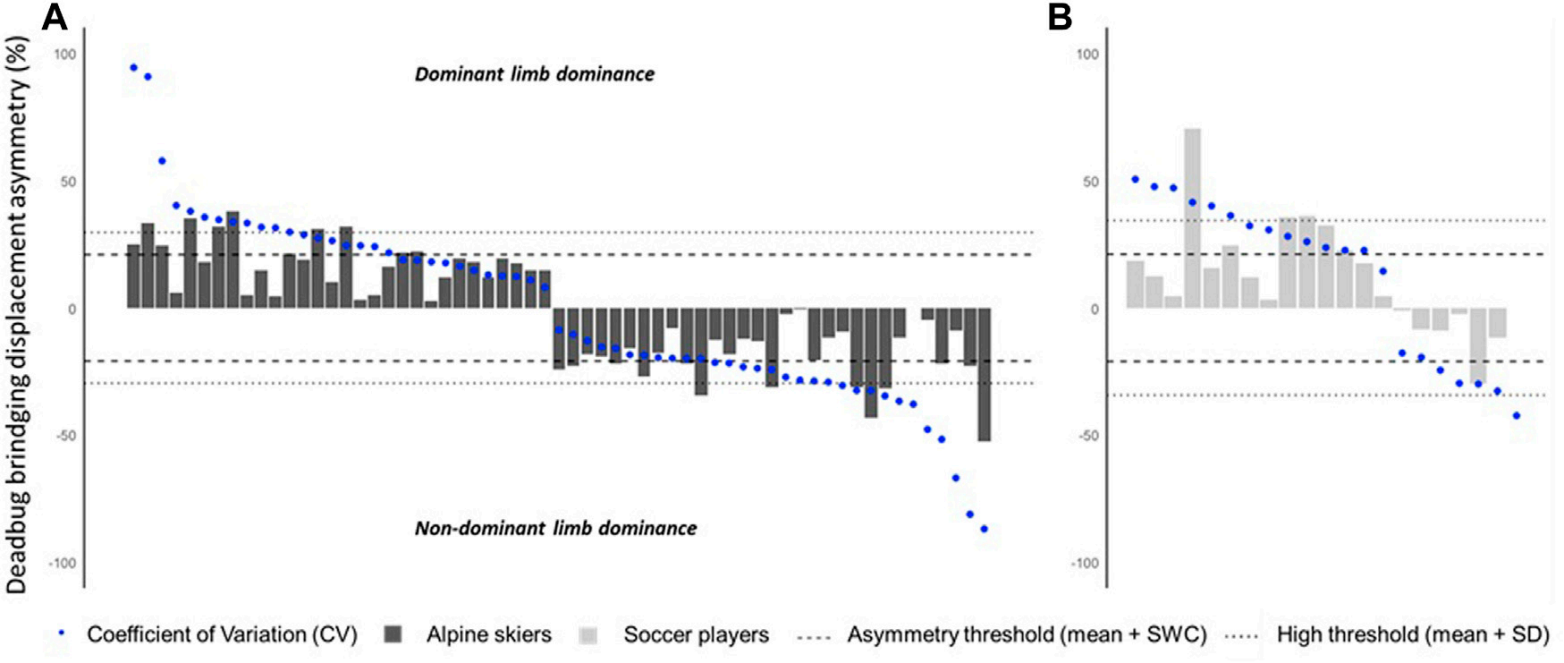
**(A,B)** Individual deadbug bridging displacement (DBB_displacement_) asymmetry data (bars) with respective coefficients of variation (blue bullet points), thresholds for small to moderate asymmetry [population mean + smallest worthwhile change (SWC), dashed lines], and thresholds for high asymmetry (population mean + SD, dotted lines). **(A)** Alpine skiers; **(B)** soccer players.

Overall, the asymmetry CV values observed were relatively high: 0.1%–178.3% and 2.2%–94.2% for *MKD* and *DBB*_*displacement*_, respectively. The sport-specific small to moderate and high asymmetry thresholds for *MKD* were 48.28% and 66.44% for soccer players (Figure 2B) and 45.10% and 65.25% for the skier group, respectively (Figure 2A). Small to moderate *MKD* asymmetries were detected in three soccer players (14.3%) and eight skiers (13.1%). High asymmetry values regarding *MKD* were found only in one soccer player (i.e., 4.8%) and in five skiers (i.e., 8.2%). For *DBB*_*displacement*_, the sport-specific thresholds for small to moderate and high asymmetries were 21.0% and 34.3% for soccer players (Figure 3B) and 20.8% and 29.6% for skiers (Figure 3A), respectively. One soccer player (i.e., 4.8%) and seven skiers (i.e., 11.5%) had small to moderate asymmetries, and three soccer players (i.e., 14.3%) and six skiers (i.e., 9.8%) had high asymmetries.

## 4 Discussion

### 4.1 Similar absolute values and asymmetry magnitudes of dynamic knee valgus and deadbug bridging performance in youth soccer players and alpine skiers

Overall, the current study revealed no significant differences in *MKD* and *DBB*_*displacement*_ between soccer players and skiers or between their dominant and non-dominant sides, underpinning the comparability of the two distinct cohorts examined in terms of their absolute values and asymmetry magnitudes of dynamic knee valgus and deadbug bridging performance. This is, on the one hand, certainly surprising, as soccer is, when compared to alpine skiing, a sport of a rather asymmetric nature (Rouissi et al., 2016; Bishop et al., 2021). However, on the other hand, a previous study in alpine skiing has reported a clear side dominance in the occurrence of ACL injuries (Westin et al., 2018), which is why the presence of lateralities in terms of functional performance factors also appears quite plausible. A potential relationship between these factors has been demonstrated, for example, for side-to-side differences in the side hop test and knee joint laxity, which have been shown to be factors that predispose skiers to ACL re-injury (Westin et al., 2022a).

### 4.2 Opposite laterality directions in soccer players compared with alpine skiers and their implications for dealing with asymmetric athletes

Despite a lack of main effects, the MANOVA revealed a crossover interaction between side* and sports for both *MKD* and *DBB*_*displacement*_. This means that there is a distinct effect of the specific sport on comparisons between the dominant and non-dominant sides.

In soccer players, *MKD* laterality was directed to the non-dominant side and *DBB*_*displacement*_ laterality to the dominant side, whereas this pattern was reversed in alpine skiers, even though it was much less pronounced. For soccer players, this implies that while DJ landing the non-dominant leg (i.e., the standing leg) expressed higher magnitudes of dynamic knee valgus than the dominant leg (i.e., the kicking leg), and during the execution of the deadbug bridging exercise, the dominant side (i.e., the side ipsilateral to the kicking leg) had poorer stabilization performance than the contralateral side. From a functional perspective, such laterality makes absolute sense, since during cutting maneuvers, high dynamic valgus loads are evoked (McLean et al., 1998; Besier et al., 2001) and soccer players can typically perform faster cutting with the dominant leg (Rouissi et al., 2016). Thus, the dominant leg is the one that has to sustain the highest valgus stress and is therefore plausible to develop higher leg axis stability over time. This also reflects muscle strength assessments, where knee flexors and extensors of the dominant leg shows superior strength capacity when compared to the non-dominant leg (Rouissi et al., 2016; Rouissi et al., 2018). Moreover, higher strength in valgus antagonistic hip abductor muscles (i.e., M. gluteus medius and minimus) was found for the dominant legs of soccer players than for the non-dominant leg (Rouissi et al., 2016; Rouissi et al., 2018). Then, while kicking, when similarly executing a deadbug bridging exercise, the dominant side (i.e., the side ipsilateral to the kicking leg) rotates around the non-dominant side (with the fixed standing leg) (Kellis and Katis, 2007; Lees et al., 2010). The corresponding rotational acceleration of the pelvis on the dominant side by the diagonal concentric activation of the obliquus internus abdominis and obliquus externus abdominis muscles represents the same activation pattern as eccentrically breaking the hip axis drop on the non-dominant side with the punctum fixum on the dominant side. Accordingly, the finding of a better deadbug bridging stabilization performance on the non-dominant side is also a plausible functional adaptation to typical loading patterns in soccer.

By contrast, *MKD* in alpine skiers again shows less pronounced but oppositely directed laterality. This may represent a slightly more symmetric stabilization capacity but with slightly higher medial displacement within the dominant leg. Likewise, *DBB*_*displacement*_ laterality was smaller than that in soccer players and oppositely directed to the non-dominant side. Another interesting observation, however, is the observation that at the individual level, a slightly higher percentage of alpine skiers were classified as asymmetric when compared to soccer players. This may be explained by the slightly lower professionalization of youth development programs at the U16 level in alpine skiing and lower financial resources that can be invested in systematically testing and addressing functional asymmetries, a difference that gradually disappears at higher levels.

In summary, despite similar absolute values and asymmetry magnitudes of dynamic knee valgus and deadbug bridging performance in youth soccer players and alpine skiers, the effect on the direction of laterality was opposite. It appears that sport-specific demands influence the direction rather than the presence and magnitude of asymmetries.

### 4.3 Study limitations

The first limitation of this study is that the coefficient of variance calculations for the *MKD* measures consisted of only two measurements, potentially limiting the representativeness for the corresponding asymmetry threshold derivation. Worth noting in this context are the rather large within-subject CV values observed in the current study. To some degree, this might be favored by the highly dynamic nature of the assessed movement tasks and also by the limited number of measurement repetitions underlying these calculations. The second limitation is differences in the number of participants within the groups, which was caused by the availability of the corresponding athletes.

## 5 Conclusion

As shown in this study, a certain degree of laterality is present in both youth soccer players and alpine skiers, and this is of a similar magnitude. However, despite similar absolute values and asymmetry magnitudes of dynamic knee valgus and deadbug bridging performance in youth soccer players and alpine skiers, the effect on the direction of laterality was opposite. This implies that the corresponding sports had a significant impact on the comparison between the dominant and non-dominant sides. Accordingly, our results underline the evident need to analyze lateralities on the basis of sport- or population-specific thresholds. Depending on the sport, laterality is not “unfavorable” *per se*, and potential functional advantages and disadvantages should be considered when addressing individual asymmetries in athletes.

## Data Availability

The datasets presented in this article are not readily available because their access is restricted to protect the interests of the project partner FC Basel and Swiss-Ski and their athletes. Requests to access the datasets should be directed to joerg.spoerri@balgrist.ch.
